# Supplementary material for: Parents’ work–family conflict and parent‒child relationship: The mediating role of parenting burnout and the moderating role of self-compassion
Source: PLoS One. 2025 Mar 18;20(3):e0319675. doi: 10.1371/journal.pone.0319675 (PMC11918315; doi:10.1371/journal.pone.0319675)
Supplement: Attachment — Submitted filename: Response to Reviewers.doc [file pone.0319675.s003.doc]

Dear reviewers,

Thank you for your email and for the opportunity to revise our manuscript titled "Parents’ work–family conflict and parent‒child relationship: The mediating role of parenting burnout and the moderating role of self-compassion" (PONE-D-24-51556), which is currently under consideration for publication in *PLOS ONE*.

We sincerely appreciate the time and effort that you and the reviewers have dedicated to providing comprehensive feedback on our work. We have carefully considered all the comments and suggestions and have made corresponding revisions to the manuscript. Below, we provide point-by-point responses to the comments, along with explanations of the changes implemented in the revised manuscript. We hope that these revisions adequately address the concerns raised. The main corrections to the paper and our responses to the reviewers’ comments are as follows.

**Point-by-point response to reviewer comments:**

To Reviewer #1:

Reviewer comment: 1. Heading "1.1 The Current Study": I believe it would be more appropriate for this section to be titled "Aim of the Study" and placed underneath the "Materials and Methods" section. This would align better with the logical flow of the manuscript.

Response: Thank you very much for your insightful comment. We fully agree with your suggestion regarding the title and placement of this section. To align better with the logical flow of the manuscript, we have made the adjustment that you recommended. The section previously titled "1.1 The Current Study" has been renamed "Aim of the Study" and moved to the "Materials and Methods" section. We believe that this change will enhance the clarity and coherence of our manuscript. Thank you again for your valuable feedback.

Reviewer comment: 2. Methods Section: As per the journal guidelines, the heading title of the "Methods" section should be revised to "Materials and Methods."

Response: Thank you for bringing this to our attention. We apologize for this oversight and have revised the heading title of the section in accordance with the journal guidelines. The "Methods" section has been renamed to "Materials and Methods" as required. We appreciate your guidance in ensuring that our manuscript adheres to the journal's formatting standards.

Reviewer comment: 4. Formatting: Please adjust the formatting of the sentences to ensure that they are aligned neatly with the margins of the document for consistency and a professional presentation.

Response: Thank you for your suggestion regarding the formatting of our manuscript. We understand the importance of a neat and consistent presentation. To address this, we have carefully adjusted the formatting of all sentences, ensuring that they are now aligned neatly with the document margins. This adjustment has been made throughout the entire manuscript to maintain a professional appearance and enhance readability. We appreciate your attention to detail and believe that these changes will contribute to a more polished final document.

To Reviewer #2:

Reviewer comment: Need for Correction: (1) work‒family conflict significantly and positively predicts Parent‒child relationship; in this hypothesis, is there positive or negative prediction in parent- child relationship should be clear.

Response: Thank you for pointing out the need for clarification in our hypothesis regarding the relationship between work‒family conflict and the parent‒child relationship. Our original hypothesis was that "work‒family conflict significantly and positively predicts the parent‒child relationship." However, this phrasing may have been misleading, as it could be interpreted that a higher level of work‒family conflict leads to a better parent‒child relationship, which is not our intended meaning.

What we actually mean is that work‒family conflict has a significant effect on the parent‒child relationship. Specifically, as the level of work‒family conflict increases, the quality of the parent‒child relationship tends to decrease. In other words, there is a negative correlation between work‒family conflict and the parent‒child relationship. Greater work‒family conflict is associated with more problems or challenges in the parent‒child relationship rather than improvement.

To correct and clarify this, we have rephrased the hypothesis as follows:

"Work‒family conflict significantly and negatively predicts the parent‒child relationship."

This revised hypothesis accurately reflects our intended meaning that increased work‒family conflict is associated with a decline in the positive aspects of the parent‒child relationship, such as reduced emotional closeness, more conflict, or less effective communication between parents and children.

We apologize for any confusion caused by the original phrasing and appreciate your feedback in helping us improve the clarity and accuracy of our research.

To Reviewer #3:

For abstract:

Reviewer comment: 1 Abstract Main Objective is missing

Response: Thank you for pointing out that the main objective of the study was missing from the abstract. We apologize for this oversight. The main objective of our study is to explore the impact of work‒family conflict on the parent‒child relationship, with a focus on the mediating role of parenting burnout and the moderating role of self-compassion. We have now included this information in the abstract to provide a clear and concise overview of the purpose of our research.

For Methodology:

Reviewer comment: Elaborate the methodology

Response: Thank you for your suggestion to elaborate on the methodology. We have supplemented the methodology section with additional details to enhance the transparency and rigor of our study.

Reviewer comment: Reliability score

Response: Thank you for your comment. The reliability scores for each of the survey questionnaires are reported in the revised manuscript. Specifically, they can be found on lines 113 (work‒family conflict), 127 (parent‒child relationship), 135 (parental burnout), and 146 (self-compassion).

For Discussion:

Reviewer comment: Check for English and grammar

Response: Thank you for your valuable feedback and for pointing out the potential language issues in our manuscript. In response to your comment, we have meticulously checked the discussion section for English and grammar to increase its readability and ensure that it meets the standards of academic writing.
