# Supplementary material for: Parents’ work–family conflict and parent‒child relationship: The mediating role of parenting burnout and the moderating role of self-compassion
Source: PLoS One. 2025 Mar 18;20(3):e0319675. doi: 10.1371/journal.pone.0319675 (PMC11918315; doi:10.1371/journal.pone.0319675)
Supplement: Attachment — Submitted filename: PLOS one.docx [file pone.0319675.s001.docx]

Comments

| Sl. No | Contents | Comments |
| --- | --- | --- |
| 1 | Abstract | Main Objective is missing |
| 2 | Introduction | No comments |
| 3 | Methodology | Elaborate the methodology  Reliability score |
| 4 | Result | No comments |
| 5 | Discussion | Check for English and grammar |

Over all check for English and grammar
